# Study of the effect of C1-esterase inhibitor administration to the sepsis pig model

**DOI:** 10.1186/cc12985

**Published:** 2013-11-05

**Authors:** Hisashi Imahase, Yuichiro Sakamoto, Taku Miyasho, Kazuto Yamashita, Jun Tamura, Takaharu Itami, Tomohito Ishizuka, Yoshio Kawamura, Tadashi Sano, Satoshi Inoue

**Affiliations:** 1 Emergency and Critical Care Center, Saga University Hospital, Saga City, Japan; 2 Department of Veterinary Science, Rakuno Gakuen University, Ebetsu, Hokkaido, Japan; 3 Department of Veterinary Medicine, Rakuno Gakuen University, Ebetsu, Hokkaido, Japan

## Background

New therapy is required that improves the prognosis of patients suffering from severe sepsis or septic shock. C1-esterase inhibitor (C1-Inh) was introduced in clinical medicine for patients with hereditary angioedema. Some studies show that C1-Inh administration may also have a beneficial effect in other clinical conditions such as sepsis [1,2]. We examined the effect of C1-Inh administration to the sepsis pig model.

## Materials and methods

The experiments were performed divided into two groups: the treatment group and the control group. We administered LPS (40 μg/kg) to pigs of about 10 kg over 30 minutes. At the same time, we administered C1-Inh in the control group (500 U, *n *= 3; 1,000 U, *n *= 3), and saline in the control group (*n *= 3). We examined the effect of C1-Inh for the outcome of the two groups, physiological indicators such as heart rates (HR) and mean arterial pressure (MAP), and autopsy results such as pleural effusion and ascites.

## Results

The outcome of the two groups was that 5/6 in the treatment group and 2/3 in the control group survived at 240 minutes from the end of LPS administration. HR (/minute) at 180 minutes from the end of LPS administration was 157.5 ± 12.3 in the treatment group and 205.3 ± 42.6 in the control group, and MAP (mmHg) at the same time was 60.0 ± 8.2 in the treatment group and 58.3 ± 5.6 in the control group. As for the autopsy results, pleural effusion (ml) was 13.28 ± 3.13 in the treatment group and 9.87 ± 4.33 in the control group, and ascites (ml) was 165.8 ± 32.99 in the treatment group and 210.0 ± 60.8 in the control group. Seeing each individual, the individual showing a large effect of C1-inh was observed.

## Conclusions

C1-Inh tended to stabilize the hemodynamics of the sepsis pig model, but was not able to reduce significantly the amount of pleural effusion and ascites.
